# Therapy results in pediatric Hodgkin lymphoma — does less mean better? Experience from a single children’s oncology center

**DOI:** 10.1007/s00277-023-05268-5

**Published:** 2023-05-17

**Authors:** Joanna Stankiewicz, Andrzej Kołtan, Ewa Demidowicz, Natalia Bartoszewicz, Sylwia Kołtan, Krzysztof Czyżewski, Monika Richert-Przygońska, Robert Dębski, Monika Pogorzała, Barbara Tejza, Joanna Cisek, Piotr Księżniakiewicz, Agnieszka Jatczak-Gaca, Agata Marjańska, Marlena Salamon, Anna Dąbrowska, Anna Urbańczyk, Elżbieta Grześk, Kamila Jaremek, Monika Łęcka, Oliwia Grochowska, Jan Styczyński

**Affiliations:** Department of Pediatric Hematology and Oncology, Collegium Medicum, Nicolaus Copernicus University Toruń, Antoni Jurasz University Hospital No.1, Ul. Sklodowskiej-Curie 9, 85-094 Bydgoszcz, Poland

**Keywords:** Hodgkin lymphoma, Children, Therapeutic era, Survival, Radiotherapy

## Abstract

Therapy results in pediatric Hodgkin lymphoma reflect remarkable progress in pediatric oncology. In the last decade, relevant development of new therapeutic options for children with refractory or relapsed disease has been made. In this study, we retrospectively analyzed therapy results and risk factors in children treated in a single oncology center according to five therapeutic protocols. Data from 114 children treated by a single institution between 1997 and 2022 were analyzed. Classic Hodgkin lymphoma therapy results were divided into four therapeutic periods: 1997–2009, 2009–2014, 2014–2019, and 2019–2022. For nodular lymphocyte-predominant Hodgkin lymphoma, data from one therapeutic protocol was analyzed. For the entire group, the 5-year probability of overall survival was 93.5%. There were no statistically significant differences between therapeutic periods. The occurrence of B symptoms at diagnosis and incidence of relapse were risk factors for death (*p* = 0.018 and *p* < 0.001). Relapse occurred in 5 cases. The 5-year probability of relapse-free survival for the entire group was 95.2%, without significant differences between groups. Patients treated between 1997 and 2009 had over a sixfold higher risk for events, defined as primary progression, relapse, death, or incidence of secondary malignancies (OR = 6.25, *p* = 0.086). The 5-year probability of event-free survival for all patients was 91.3%. Five patients died, and the most common cause of death was relapse. Modern therapeutic protocols in pediatric Hodgkin lymphoma are marked by excellent outcomes. Patients with disease relapses have a notably high risk of death, and the development of new therapeutic options for this group remains one of the main goals of current trials.

## Introduction

Hodgkin lymphoma (HL) represents about 5–7% of all malignancies in children, with an incidence of 5:100,000 per year in children up to 15 years old [1]. The etiology of the disease is not well understood, but there is growing evidence of an Epstein-Barr infection in the pathogenesis of HL. Moreover, patients with immunodeficiencies or on immunosuppressive therapy have an increased risk of HL [1, 2]. The pathomorphological classification divides HL into classical HL with four subtypes (nodular sclerosis, mixed cellularity, lymphocyte rich, and lymphocyte depleted) and nodular lymphocyte-predominant HL (nLPHL) type, which represent about 10% of all new cases. Treatment of HL consists of chemotherapy combined with radiotherapy in most cases. During the last few years, new therapeutic options for children with targeted agents and immunotherapy have been under investigation in clinical trials [2–4].

Therapy results in pediatric HL have made remarkable progress in the treatment of childhood malignancies. Whereas the 5-year probability of overall survival (pOS) exceeded 90% over 20 years ago, further efforts have focused on reducing side effects, especially the late ones [5, 6]. In 1997, the Polish Pediatric Leukemia and Lymphoma Study Group introduced the first unitized therapy protocol for newly diagnosed children with HL in Poland. The aim of the protocol was not only to improve therapy results but also to minimize long-term complications [7]. Since 2007, pediatric oncology centers in Poland have been part of the international EuroNet-PHL trials that have focused on therapy individualization according to risk group stratification and treatment response [8].

In this study, we analyzed therapy outcomes in children with HL treated in a single oncology center in Poland over a period of 25 years.

## Materials and methods

### Design of the study

Retrospective analysis of therapy results and risk factors in children treated for HL in a single oncology center according to different therapy protocols.

### Patients

In the study, data from children aged 3.5–20.8 years, treated by the Department of Pediatrics, Hematology and Oncology in Bydgoszcz during 1997–2022, were analyzed. The inclusion criteria involved children with a confirmed diagnosis of HL, both classical HL (nodular sclerosis, mixed cellularity, lymphocyte rich, and lymphocyte depletion subtypes) as well as nLPHL. Patients with incomplete data, as well as patients treated according to the EuroNet-PHL-C2 clinical trial (ClinicalTrials.gov identifier: NCT02797717), were excluded, as the results of that study have not been published yet.

### Diagnosis

The diagnosis of HL was established according to pathology results. A detailed medical interview focusing on the B symptoms (unexplained fever, weight loss, and night sweats) was performed in each case. Complete blood count, lactate dehydrogenase (LDH) level, erythrocyte sedimentation rate (ESR), ultrasonography (USG) of the lymph nodes, liver, and spleen, and echocardiography (ECG) were performed in all cases. Bone marrow trephine biopsy was performed up to 2009. All patients also had computer tomography (CT) scans performed on the neck, chest, abdomen, and pelvis. Since 2009, whole-body positron emission tomography-CT (PET-CT) scans were performed.

Lymph nodes were considered involved in cases of enlargement above 2 cm between 1997 and 2009. Starting in 2009, all patients had a whole body PET-CT scan, and lymph nodes were defined as involved in cases with a diameter of 1–2 cm with a positive PET-CT or a diameter exceeding 2 cm independent of PET-CT results.

The lymph regions involved were scored based on the Ann Arbor classification in Cotswold modification [9].

Bulk was defined as present if the volume of the largest contiguous lymph node mass was ≥ 200 mL. Spleen involvement occurred when the spleen was PET-CT positive or multiple small focal changes were detected on USG. Lung involvement was diagnosed in cases of 2 or more lesions with a diameter of 2–10 mm or at least one lesion ≥ 10 mm in diameter. Liver involvement was defined as PET-CT positive changes or a minimum of one focal change detected with other imaging methods. Bone marrow involvement was defined as the presence of lymphoma cells in one marrow biopsy before 2009 and more than two PET-CT positive bone lesions after 2009. Bone involvement was detected based on CT scans of the bones. Extra-lymphatic structures or organs infiltrated per continuum were termed E-lesions.

Between 1997 and 2009, the total volume of involved lymph nodes was calculated, followed by the formula that lymph nodes of a diameter below 2 cm were equal to 1 unit, between 2 and 5 cm — 2 units, and above 5 cm — 3 units. In cases of mediastinal involvement, if the largest diameter of lymphoma mass was lower than 1/4 diameter of the chest, it was equal to 1 unit, 1/4–1/3 of the chest was equal to 2 units, and more than 1/3 of the chest — 3 units. In cases of bilateral mediastinal involvement, the unit numbers doubled [7].

### Therapy protocols and risk group stratification

Children have been treated according to consecutive therapeutic protocols: PGP-HD-97 from January 1997 to June 2009 [7]; EuroNet-PHL-C1 from July 2009 to February 2014 [8]; EuroNet-PHL-Interim Phase from March 2014 to September 2019, and EuroNet-PHL-C2 from October 2019 [10]. Patients diagnosed with nLPHL were treated according to the Euro-Net-PHL-LP1 protocol (ClinicalTrials.gov identifier: NCT01088750).

**Fig. 1:**
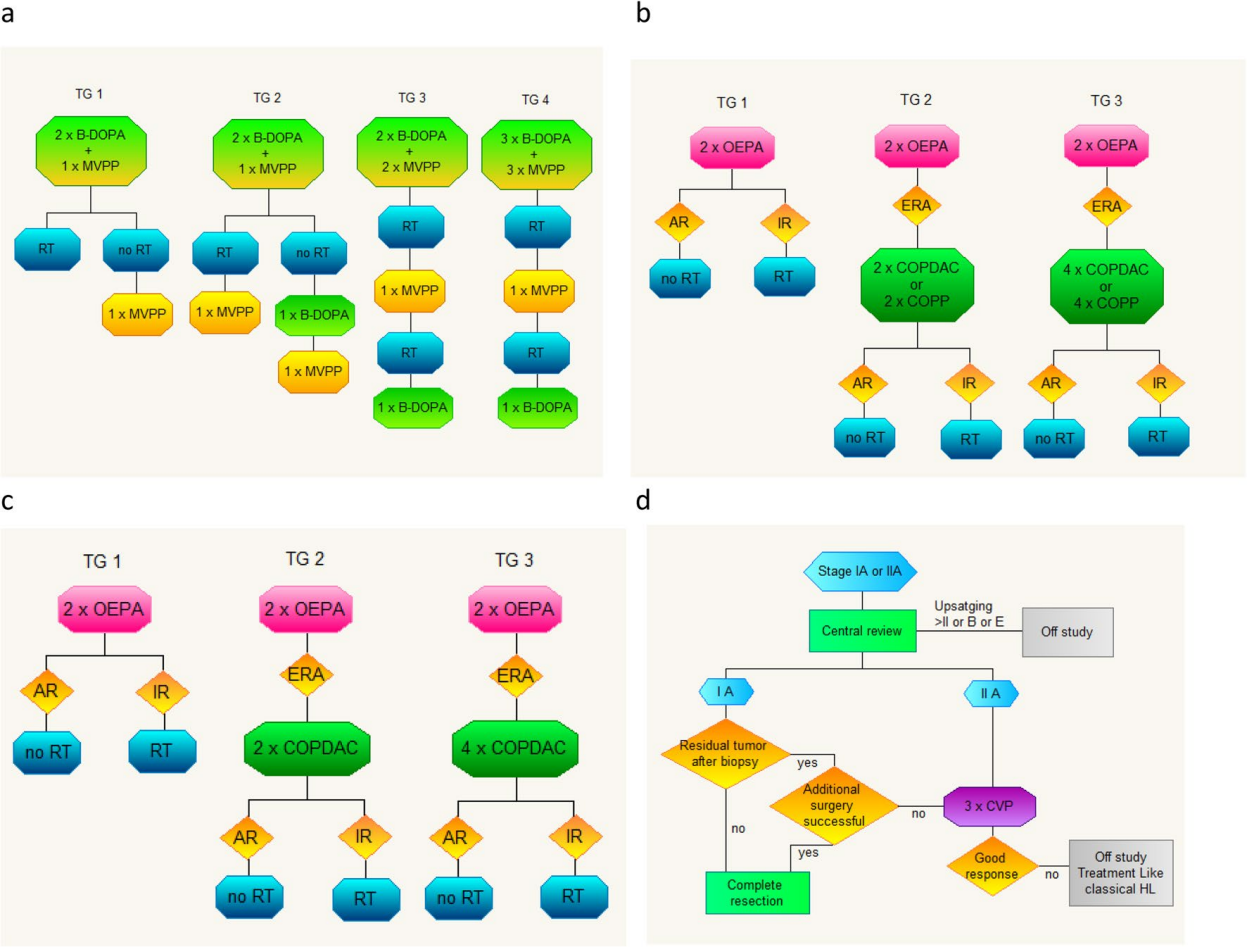
Schemas of four consecutive protocols analyzed in the study. **a** PGP-HD-97 protocol; **b** EuroNet-PHL-C1 protocol; **c** EuroNet-PHL-Interim protocol; **d** Euro-Net-PHL-LP1 protocol; TG 1 — therapeutic group 1; TG 2 — therapeutic group 2; TG 3 — therapeutic group 3; TG 4 — therapeutic group 4; B-DOPA — bleomycin, dacarbazine, vincristine, doxorubicin, prednisone; MVPP – nitrogranulogen, vinblastine, procarbazine, prednisone; RT — radiotherapy; OEPA — vincristine, etoposide, prednisone, doxorubicin; AR — adequate response; IR — inadequate response; ERA — early response assessment; COPP — cyclophosphamide, vincristine, procarbazine, prednisone; COPDAC — cyclophosphamide, vincristine, prednisone, dacarbazine; DECOPDAC — dacarbazine, etoposide, doxorubicin, cyclophosphamide, vincristine, prednisone; B — B symptoms, E — E-lesions; CVP — cyclophosphamide, vinblastine, prednisone

Protocol schemes are shown in Fig. 1.

During the PGP-HD-97 therapeutic period, patients were stratified into 4 risk groups, according to lymph node regions involved and the presence of B symptoms, defined as below:

- Group 1 — Stages IA and IB (with the exception of the lymphocyte depletion subtype).
- Group 2 — Stage IIA in children ≤ 10 years old and a total lymph node mass below 8 units.
- Group 3 — Stage IIA in children > 10 years old and total lymph node mass 8 units and above or stages IIB and IIIA (both with the exception of the lymphocyte depletion subtype).
- Group 4 — Stages IIIB and IV, and all stages of lymphocyte depletion subtype.

According to EuroNet-PHL-C1, EuroNet-PHL-interim phase, and EuroNet-PHL-C2 patients were stratified into 3 therapeutic groups (TG):

- TG1 — Stages IA and IIA without risk factors defined as ESR ≥ 30, missing ESR, or occurrence of bulk.
- TG2 — Stages IA and IIA with at least one risk factor or stage IIB and IIIA without E-lesions.
- TG3 — Stages IIB and IIIA with E-lesions, or stages IIIB or IV.

Euro-Net-PHL-LP1 was dedicated to children with nLPHL in stages IA and IIA, and it did not define specific therapeutic groups. The protocol offered two paths of treatment, one for stage IA and a second one for stage IIA.

### Response criteria

Response to initial therapy was evaluated after 3 cycles of chemotherapy during the PGP-HD-97 protocol and was defined as adequate in cases of at least 70% reduction of the primary lymphoma mass on CT.

In EuroNet PHL-C1, EuroNet-PHL-Interim Phase, and EuroNet PHL-C2 trials, the response was evaluated after 2 cycles of chemotherapy using CT or MRI and fluorodeoxyglucose (FDG)-PET scan results. The response was defined as adequate, if the response assessment PET was negative or the residual tumor volume was less than or equal to 5% of the reference volume and less than or equal to 2 ml, or all disease symptoms disappeared and the response assessment PET was unclear.

During Euro-Net-PHL-LP1, the response was assessed after 3 cycles of chemotherapy in stage IIA or just after initial surgery in stage IA according to CT or MRI and FDG-PET scan results. Complete remission was defined as the disappearance of all disease symptoms, non-measurable tumor volumes in all initially involved regions, and negative results on the response assessment PET scans.

### Definitions

Progression was defined as the recurrence or occurrence of new disease symptoms which could not be explained otherwise, the occurrence of new lymphatic or extra-lymphatic lesions, or at least one initially involved extra-nodal site or region with non-measurable tumor volume was locally progressive during therapy or within 3 months after the end of therapy. Relapse was defined as the occurrence of new lymphatic or extra-lymphatic lesions or if at least one initially involved an extra-nodal site or involved a region with a non-measurable tumor volume that progressed 3 months after the end of therapy or further. An event was defined as primary progression, relapse, death of any causes, or incidence of secondary malignancies. Overall survival was defined as the time from HL diagnosis until death of any cause or end of follow-up. Relapse-free survival (RFS) was defined as the time from HL diagnosis until a relapse occurred. Event-free survival (EFS) was defined as the time from diagnosis to event occurrence.

### Statistical analysis

The endpoints of the study included a 5-year pOS, a 5-year probability of EFS (pEFS), and a 5-year probability of RFS (pRFS). Survival curves of pOS, pEFS, and pRFS were calculated using the Kaplan–Meier method and compared by log-rank tests. All clinical, laboratory, and imaging data were analyzed in terms of their impact on outcomes. Univariate analysis was used to assess prognostic factors. The significant factors from univariate analysis were used in the multivariate regression model. Data were considered statistically significant when the *p* value was < 0.05. All calculations were performed using the statistical software MedCalc 20.100 (MedCalc Software, Mariakerke, Belgium). The study was approved (KB 577/2021) by the Ethics Committee of Collegium Medicum, Nicolas Copernicus University, Bydgoszcz.

## Results

A total of 123 patients were treated for HL between April 1997 and March 2022. Five patients were excluded because of insufficient or missing data and another four were excluded because they were part of the EuroNet-PHL-C2 clinical trial. Data from 114 children were analyzed. Fifty-four of them (47.4%) were male and 60 (52.6%) were female (M/F 1:1.1). The mean age at diagnosis was 14.1 years and the median age was 15.3 years. Over 90% of patients had more than 10 years at diagnosis. The most common manifestation of the disease was a mediastinal mass (89 patients, 78.1%), followed by cervical lymphadenopathy (87 patients, 76.3%) and supraclavicular lymph node enlargement (84 patients, 73.7%). All three sites were involved in over half of the cases (*n* = 61, 53.4%). B symptoms occurred in 52 children (45.6%). E-lesions were observed in 7 cases (6.1%), bulky disease in 24 (21.1%), 18 patients (15.8%) had lung involvement, and another 12 (10.5%) had skeletal involvement.

One hundred and eleven patients were diagnosed with classical HL and three (2.6%) with nLPHL. In the group of classical HL, the most frequent histological subtype was nodular sclerosis (*n* = 69, 62.1%), followed by mixed cellularity (*n* = 18, 16.2%). Two patients had the nodular predominant subtype and another two lymphocyte rich (1.8% in each case). The characteristics of patients from consecutive therapeutic protocols are shown in Table 1.

**Table 1:** The characteristics of patients from consecutive therapeutic protocols

	PGP-HD-97	EuroNet-PHL-C1	EuroNet-PHL-Interim Phase	EuroNet-PHL-C2	Euro-Net-PHL-LP1
Number of patients	51	17	27	14	3
Gender					
Male	25	8	10	5	3
Female	26	9	17	9	0
Age (years) mean range	14.1 4.1–18.8	14.4 3.9–20.8	13.7 3.5–17.7	14.2 5.7–18.2	12.6 6.8–15.7
WBC (× 10^3) mean range	10.2 2.4–33.0	11.5 6.5–21.7	10.5 4.2–18.8	11.2 4.9–27.1	8.7 7.4–9.8
PLT (× 10^3) mean range	377 153–972	349 67–646	354 178–541	424 163–792	304 222–366
HGB (g/l) mean range	11.8 8.7–16.3	11.9 8.8–16.0	11.8 8.2–14.1	11.7 7.6–15.3	13.9 13.2–14.9
ESR mean range	49 2–135	38 1–83	46 11–140	46 4–105	7 2–16
B symptoms					
Fever	7	3	8	3	0
Weight loss	17	7	3	4	0
Night sweats	10	4	10	3	0
Lymph nodes regions involvement – median range	4 1–9	5 2–7	3 1–9	5 2–7	2 1–2
Liver involvement	6	2	2	2	0
Spleen involvement	11	2	9	4	0
Skeletal involvement	3	3	5	3	0
Lung involvement	5	4	5	5	0
Bulky disease	5	2	9	3	0
E-lesions	1	6	7	0	0
Stage at diagnosis					
I	3	0	0	0	1
II	26	10	11	6	2
III	12	1	8	2	0
IV	10	6	8	6	0

*WBC* White blood cells, *PLT* platelet count, *HGB* hemoglobin

## Therapy results

Adequate response after initial therapy was observed in 62.9% of patients and for the consecutive protocols a total of 67.7% for PGP-HD-97, 53.3% for EuroNet-PHL-C1, 48.1% for EuroNet-PHL-Interim, and 60.0% for EuroNet-PHL-C2. Patients with inadequate responses to initial therapy had almost a fivefold higher risk of death, but the differences were not statistically significant (OR = 4.68, 95% Cl 0.50 to 43.12, *p* = 0.172). Fifty-eight patients (50.8%) were treated with radiotherapy.

**Fig. 2:**
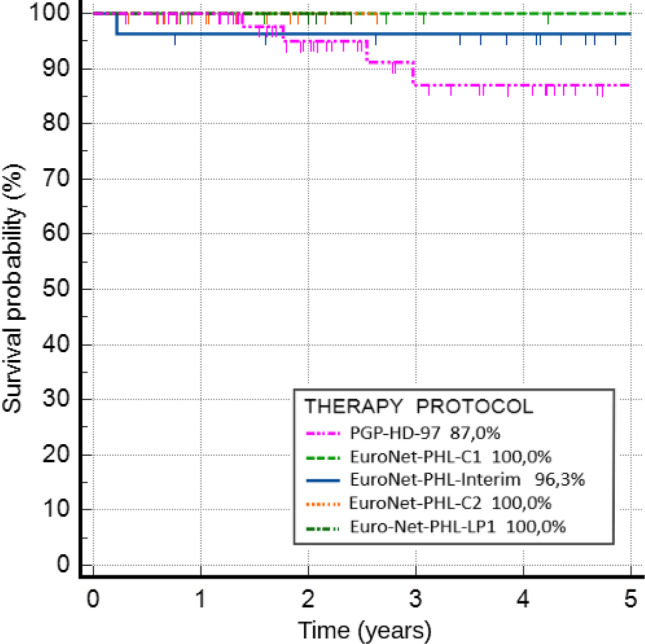
Five-year pOS for children treated because of HL between 1997 and 2022 according to 5 consecutive protocols

The 5-year pOS for the entire group was 93.5%. For each protocol, the 5-year pOS was 87.0% for PGP-HD-97, 100.0% for EuroNet-PHL-C1, 96.3% for EuroNet-PHL-Interim, 100.0% for EuroNet-PHL-C2, and 100.0% for Euro-Net-PHL-LP1. The results are shown in Fig. 2. The differences between protocols were not statistically significant (*p* = 0.602). Risk factors related to poor outcomes was the occurrence of B symptoms (5-year pOS 86.5% vs. 93.5%, *p* = 0.018). Patients treated according to PGP-HD-97 had an 11-fold higher risk of death compared to the others (OR 11.18, 95% Cl 0.62–200.31, *p* = 0.080). Outcomes for the consecutive therapeutic protocols are shown in Table 2.

**Table 2:** Outcomes of five consecutive therapeutic protocols in the analyzed group

Outcome	PGP-HD-97	EuroNet-PHL-C1	EuroNet-PHL-interim phase	EuroNet-PHL-C2	Euro-Net-PHL-LP1
Primary progression	1	1	0	0	0
Relapse	4	0	0	1	0
Secondary malignancy	1	0	0	0	0
Death	4	0	1	0	0
Reason of death					
Relapse	3	0	0	0	0
Treatment-related mortality	0	0	1	0	0
Secondary malignancy	1	0	0	0	0

**Fig. 3:**
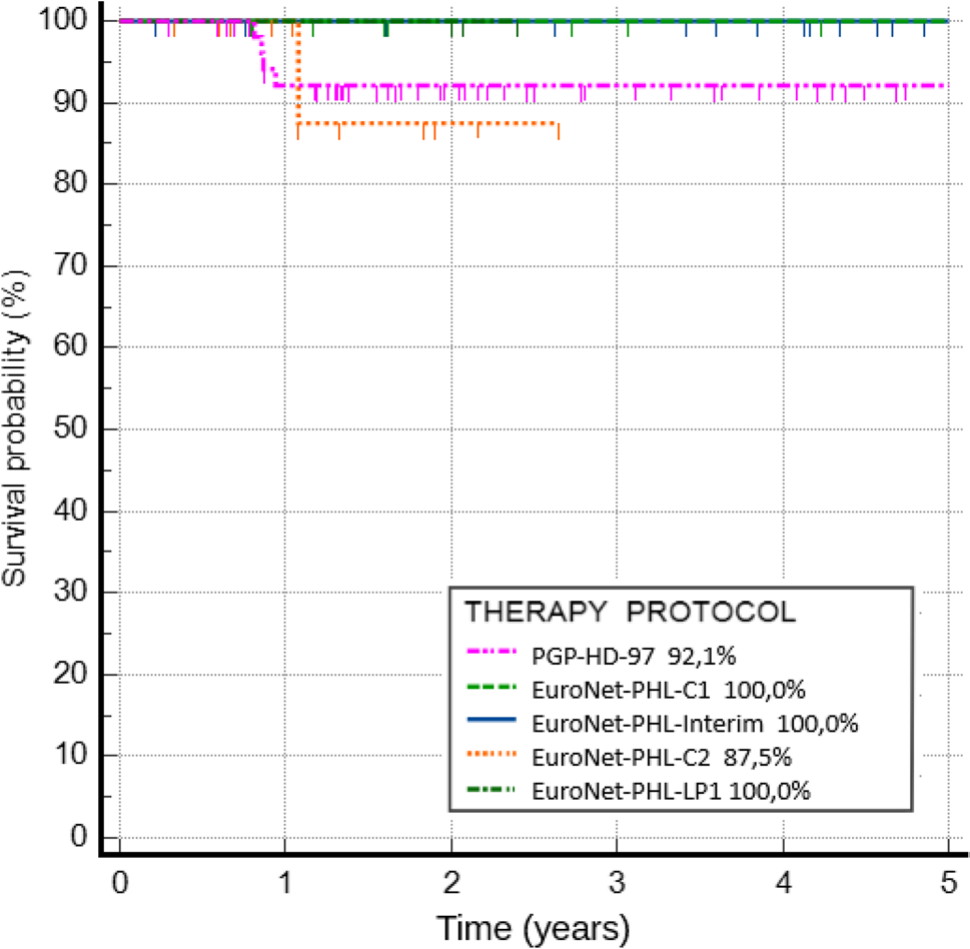
Five-year pRFS for children treated because of HL between 1997 and 2022 according to 5 consecutive protocols

Relapse occurred in 5 cases with a median time to relapse of 0.86 years. In five patients with relapse, second-line therapy consisted of chemotherapy with autologous hematopoietic cell transplantation (auto-HCT) in two patients, chemotherapy only in one patient, and chemotherapy and radiotherapy in one patient. One patient was treated according to the EuroNet-PHL-C2 protocol after a 2020 relapse due to early therapy termination because of treatment toxicity. In this case, the first chemotherapy cycle was complicated with *Clostridium perfringens* sepsis that required treatment in the intensive care department. After he recovered from sepsis, the treatment was continued, but after a few days, he presented severe bleeding from the gastrointestinal tract, which was complicated by sudden cardiac arrest with successful resuscitation. Because of the severe adverse effects of chemotherapy, he was switched to second-line therapy with brentuximab vedotin (BV) in combination with nivolumab. He is still completing therapy with good clinical and radiological responses. Three patients died after relapse. The 5-year pRFS for the entire group was 95.2%. For each protocol, the 5-year pRFS was 92.1% for PGP-HD-97, 100.0% for EuroNet-PHL-C1, 100.0% for EuroNet-PHL-Interim, 87.5% for EuroNet-PHL-C2, and 100.0% for Euro-Net-PHL-LP1. The results are shown in Fig. 3. There were no statistically significant differences between protocols and risk groups (*p* = 0.277, *p* = 0.763). Platelets count above 450 × 10^9/L at diagnosis was an independent risk factor for relapse (*p* = 0.013). The incidence of relapse was the most important risk factor for death (*p* < 0.001).

**Fig. 4:**
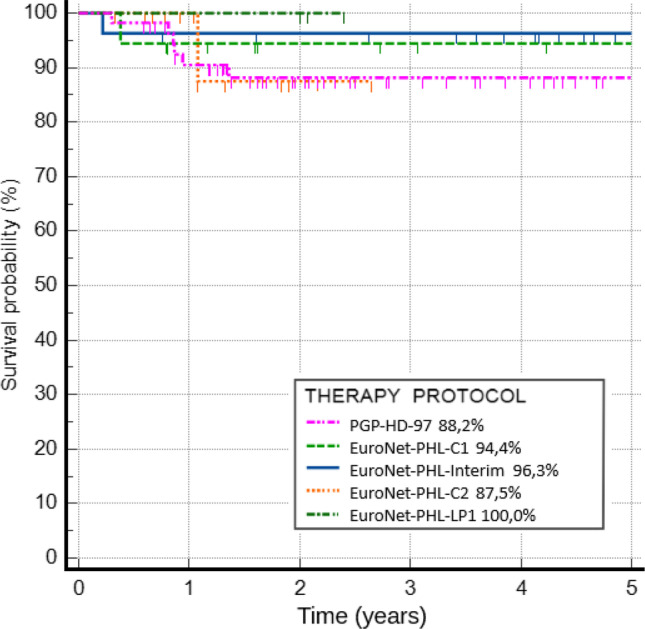
Five-year pEFS for children treated because of HL between 1997 and 2022 according to 5 consecutive protocols

The 5-year pEFS for all patients was 91.3%. For each protocol, the 5-year pEFS was 88.2% for PGP-HD-97, 94.4% for EuroNet-PHL-C1, 96.3% for EuroNet-PHL-Interim, 87.5% for EuroNet-PHL-C2, and 100.0% for Euro-Net-PHL-LP1, without statistically significant differences between protocols (*p* = 0.721). The results are shown in Fig. 4. There were no differences in event occurrences between risk groups (*p* = 0.907). However, therapy with the PGP-HD-97 protocol was related to considerably poorer outcomes with an over sixfold higher risk of events (OR = 6.25, 95% CI 0.76–50.84, *p* = 0.086). Primary progression occurred in two patients, and both of them were successfully treated with second-line therapy (chemotherapy only in one case, chemotherapy with auto-HCT in the other). Secondary malignancy occurred in one girl treated primarily with chemotherapy and radiotherapy, who was diagnosed with acute myelocytic leukemia 0.6 years after she had finished HL treatment.

## Death

During the entire analysis period, 5 patients died. The mean time from diagnosis to death was 1.54 years (median 1.57 years, range 0.22 to 2.97 years). Two patients died of relapse and one of a secondary malignancy. Among patients who died of a relapse, the second-line therapy differed significantly: the first patient was treated with etoposide, ifosfamide, and prednisone (IEP) and vindesine, lomustine, and melphalan (CAD) chemotherapy combined with radiotherapy and the second one with IEP and ifosfamide, vinorelbine, gemcitabine, and prednisone (IGEV) chemotherapy followed by auto-HCT, and the third one with IEP and doxorubicin, bleomycin, vinblastine, and dacarbazine (ABVD) chemotherapy followed by auto-HCT. There was also one case of treatment-related mortality because of an infection during chemotherapy. The most important risk factor for death was the incidence of relapse (*p* < 0.001).

## Discussion

During the last 30 years, treatment results in pediatric HL have improved significantly [1]. Despite great progress in cure rates, side effects of therapy remain a relevant problem. Long-term observations have proved patients treated in childhood for HL are at risk of long-term complications, including gonadotoxicity, cardiovascular complications, thyroid dysfunctions, and secondary malignancies [11]. One of the milestones of the current therapeutic approach is to reduce radiotherapy in patients who can be treated satisfactorily with chemotherapy alone.

In Poland, the PGP-HD-97 protocol was the first protocol to omit radiotherapy in specific groups of patients [7]. The aim of radiotherapy exclusion was to protect children from the late side effects, especially cardiotoxicity, as well as thyroid and lung damage [7]. In a nationwide analysis, the 5-year pOS, pEFS, and pRFS were 92%, 96%, and 97%, respectively [7]. Results from our center were slightly inferior to the national rates, mostly due to a high rate of death due to relapse, progression, and treatment-related toxicities. Although there were no statistically significant differences in the outcomes between individual protocols, patients treated according to the PGP-HD-97 protocol had a higher risk of events compared to patients treated according to the EuroNet-PHL trials. Moreover, the number of patients with late therapy complications remained high, despite the decreased number of children who received radiotherapy during treatment [12].

Encouraging results from the Multicenter Trial GPOH-HD 95 and GPOH-HD-2002 Study resulted in the multinational EuroNet-PHL-C1 randomized trial [8, 13, 14]. This study started in January 2007 and was introduced in our department in July 2009. The treatment was based on response-adapted therapy, measured with PET with radiotherapy omission in patients who had an adequate response to the induction. The second objective of the trial was to replace procarbazine with dacarbazine during consolidation to reduce gonadotoxicity. Results of the EuroNet-PHL-C1 protocol in the analyzed group were excellent, with a 5-year pOS of 100.0% and a 5-year pEFS of 96.3%. During this therapeutic period, there was only one case of primary progressive disease, successfully treated with second-line therapy. The followed therapeutic EuroNet protocols have shown similar satisfying results in our cohort.

In adult patients, the role of FDG-PET in the response assessment is well established [15]. It has also been proved that the use of FDG-PET in an initial evaluation and response assessment has better specificity than CT alone for baseline measurements and relapse predictions [16]. The introduction of PET-based response-adapted therapy in children can help in the identification of patients with inadequate responses to initial therapy, who may benefit from additional cycles of chemotherapy or radiotherapy. It can also identify patients with a good initial response and low risk of relapse, who can be treated without radiotherapy and avoid potentially life-limiting late effects [8].

The stage at diagnosis is an important prognostic factor in HL. It was used for stratification in the EuroNet trials as well as the previous HD-97 therapeutic protocol. Additionally, in EuroNet protocols, the occurrence of B symptoms, ESR equal to or above 30 mm/h, and bulky disease were considered risk factors and stratified patients into a higher risk group [8]. In our analysis, the occurrence of B symptoms was related to a significantly lower pOS, despite being used in therapeutic group stratification. Platelet counts above 450 × 10^3/μl at diagnosis were another risk factor related to relapse. Similar observations were published by other authors [17].

The incidence of relapse was the strongest risk factor for death. Because of the long period of analysis, the therapy for relapsed disease differed widely between individual patients. From the year 2002, auto-HCT became available for relapse treatment in our patients. Although auto-HCT is the standard treatment for relapsed adult patients, there is a lack of randomized trials in children that proves the superiority of high-dose chemotherapy (HDC) with auto-HCT versus standard-dose chemotherapy (SDC) [18, 19]. Furthermore, the evidence for a survival benefit in children treated with HDC with auto-HCT is limited [19–21]. According to the EuroNet Pediatric Hodgkin Lymphoma Group’s recommendations, HDC with auto-HCT should be limited to standard and high-risk groups, while children in the low-risk group at relapse should be treated with SDC and radiotherapy [19]. Targeted agents and immunotherapy play an emerging role in relapse treatment, but their place in the treatment of relapsed/refractory HL in the pediatric population is not well specified [4, 18, 19, 22]. In our analysis, one patient was treated with BV combined with nivolumab, as his parents did not consent to chemotherapy and auto-HCT for second-line treatment. He had a complete metabolic response after 4 cycles of BV and nivolumab infusions. Although the results from trials with BV and nivolumab as second-line therapy in adults are promising, we need more information about its efficacy in children [3, 23].nLPHL represents a small subset of all HL cases in children [2]. Children with nLPHL are usually diagnosed in the early stages of the disease (IA or IIA) and have excellent outcomes with a pOS of approximately 100% [24]. Because of the indolent character of nLPHL, the therapeutic approach differs from the one used in classical HL. First-line therapy for patients with stage IA is surgery only, with further observation for patients with complete resection [2, 24]. According to the Euro-Net-PHL-LP1 protocol, 3 cycles of chemotherapy with cyclophosphamide, vincristine, and prednisone (CVP) is recommended for patients with stage IIA. In the analyzed group, there were 3 cases of nLPHL, two with stage IIA, and one with stage IA. All of them were treated with CVP chemotherapy (the patient with stage IA was because of incomplete initial resection), with a 5-year pOS, pEFS, and pRFS of 100%. These results were superior to the national analysis of the Polish Pediatric Leukemia/Lymphoma Study Group, where patients with IA and IIA stages were characterized with a 5-year pOS of 100% and a 5-year pRFS of 86.7% [25]. The prognosis for patients with stages III and IV is considerably worse than for the early stages of nLPHL, with particular concern for transformation to an aggressive, diffuse large B cell lymphoma [2].

An obvious limitation of our study is a very heterogeneous group of patients who were treated during a period of over 20 years. Due to the retrospective design, the long period of time considered during which the therapeutic protocols for pediatric HL evolved, the evaluation of disease extension at diagnosis was made in different manners, and the evaluation of response was made with different timing, methods, and criteria. Despite the effect of different therapeutic protocols, progress in supportive care as well as the development of new diagnostic tools has undoubtedly significantly impacted the outcomes. However, there were a relatively large number of cases, and despite the long period of analysis, most of the important information about clinical, laboratory, and radiological results were available. A large multicenter analysis is needed for further conclusions. We eagerly await the results of the EuroNet-PHL-C2 clinical trial.

## Conclusion

In conclusion, progress in the treatment of childhood HL is visible in both international as well as single-institution analyses. Despite some obvious limitations of our study, our analysis shows that modern therapeutic protocols are marked by excellent outcomes. Currently, the greatest emphasis is put on toxicity reduction, with radiotherapy omission as one of the core goals of new trials. Furthermore, targeted therapy and immunotherapy remain emerging therapeutic options, especially for children with relapsed or progressive disease as a group with unfavorable prognosis. Further investigation with multicenter studies is needed to confirm the initially satisfying results.

## Data Availability

Data used in the article is available from the corresponding author in case of reasonable request.
